# Does risk-adjusted payment influence primary care providers’ decision on where to set up practices?

**DOI:** 10.1186/s12913-018-2983-3

**Published:** 2018-03-14

**Authors:** Anders Anell, Margareta Dackehag, Jens Dietrichson

**Affiliations:** 1 Department of Business Administration, School of Economics and Management, Lund University, Box 7080, SE-22007 Lund, Sweden; 2 Department of Economics, School of Economics and Management, Lund University, Box 7082, SE-22007 Lund, Sweden; 3 VIVE – The Danish Center for Social Science Research, Herluf Trolles Gade 11, DK-1052 Copenhagen, Denmark

**Keywords:** Primary health care, Establishment, Equal access, Private provision, Risk-adjusted capitation, Sweden

## Abstract

**Background:**

Providing equal access to health care is an important objective in most health care systems. It is especially pertinent in systems like the Swedish primary care market, where private providers are free to establish themselves in any part of the country. To improve equity in access to care, 15 out 21 county councils in Sweden have implemented risk-adjusted capitation based on the Care Need Index, which increases capitation to primary care centers with a large share of patients with unfavorable socioeconomic and demographic characteristics. Our aim is to estimate the effects of using care-need adjusted capitation on the supply of private primary care centers.

**Method:**

We use a dataset that combines information on all primary care centers in Sweden during 2005–2013, the payment system and other conditions for establishing new primary care centers used in the county councils, and demographic, geographic, and socioeconomic variables for low-level geographic areas. To estimate the effects of care-need adjusted capitation, we use difference-in-differences models, contrasting the development over time between areas with and without risk-adjusted capitation, and with high and low Care Need Index values.

**Results:**

Risk-adjusted capitation significantly increases the number of private primary care centers in areas with relatively high Care Need Index values. The adjustment results in a changed distribution of private centers within county councils; the total number of private centers does not increase in county councils using care-need adjusted capitation. The effects are furthermore increasing over the first three years after the implementation of such capitation, and concentrated to the lower and middle range of the group of areas with high index values.

**Conclusions:**

Risk-adjusted capitation based on the Care Need Index increases the supply of private primary care centers in areas with unfavorable socioeconomic and demographic characteristics. More generally, this result indicates that risk-adjusted capitation can significantly affect private providers’ establishment decisions.

**Electronic supplementary material:**

The online version of this article (10.1186/s12913-018-2983-3) contains supplementary material, which is available to authorized users.

## Background

There are persistent socioeconomic inequalities in health and health care utilization, making equitable access to care an important policy goal [1]. Whether measured as income, education or wealth, individuals with lower socioeconomic status (SES) tend to live shorter, report worse self-assessed health and experience more chronic diseases while making fewer specialist visits, using less dental and preventive care and consuming less prescription pharmaceuticals [2–5].

One important condition for equitable access and health care utilization is the geographical distribution of providers. This is especially pertinent for primary care, as reduced access is associated with lower health care utilization and worse health [6–11]. Spatial accessibility, as defined by Guagliardo [12], comprises both the number of local providers available for the patient to choose from and the time or distance the patient needs to travel to reach a provider.

Goddard et al. [13] define three ways in which health care authorities can affect spatial accessibility: general supply increases, regulation of entry, and targeted initiatives aimed at under-supply in particular areas. Market-based entry may increase supply, but risks increasing socioeconomic inequalities in access to care if it is more profitable to be located or easy to attract staff in high SES areas. Regulated entry is a direct way to affect access, but may result in lower supply and, if providers have knowledge about local conditions that the health care authority lacks, a less efficient distribution. The third option, to target initiatives to areas where access is lower, may be a good compromise but seems to have been less evaluated so far [14, 15].

Since 2007, Swedish primary care, governed and predominantly operated by 21 elected county councils, has undergone a major change involving choice and privatization, and potentially, consequences for the equitable access to care [16, 17]. A principle of free establishment, implemented nationally in 2010, implies that county councils cannot influence private primary care centers’ decisions about where to set up their practices by means of direct regulation. Instead, many county councils have adjusted the capitation payment to primary care centers based on socioeconomic factors related to enrolled individuals. With this adjustment, county councils aim to give primary care centers a fair payment based on socioeconomic needs and to incentivize more providers to be located in geographical areas with low SES.

A recent study finds that the reforms in Swedish primary care are associated with only minor negative effects on geographical equity, despite free establishment for private primary care centers [18]. Isaksson et al. [18] identify risk-adjusted payment as a possible cause to this finding, and calls for an evaluation of the relationship between payment design and provider behavior. Heeding that call, our study investigates how risk-adjustment by socioeconomic factors influences the location decisions by newly established private primary care centers.

The empirical literature on provider behavior and payment design shows that providers generally respond to economic incentives according to theoretical expectations [19–23]. According to the standard result, fee-for-service generates high service production but low cost control, compared to capitation. However, capitation generates incentives for the provider to select “good” risks, and to overprovide services to the relatively healthy and to underprovide to the relatively unhealthy [24]. Thus, to the extent that low SES is related to greater care need (and larger costs) a simple undifferentiated capitation payment may contribute to socioeconomic differences in health care utilization. This risk highlights the importance of adjusting the capitation for expected variations in health care utilization based on health care need in the relevant patient population [25]. However, it cannot be taken for granted that primary care providers will allocate a risk-adjustment payment to its enrolled population based on health care needs. Alternatively, the additional payment based on socioeconomic factors may be used for other purposes or simply increase the profitability of services. This motivates empirical analyses of the effects of risk-adjusted payment on provider behavior, both in terms of setting up a new practice and how the additional payment influences service provision. In this paper we study how risk-adjusted payment influences decisions on where to set up a new practice.

### Swedish primary care, the patient choice reforms and risk-adjusted payment

The Swedish primary care belongs to a healthcare system characterized by publicly financed and predominantly publicly produced health care. By law primary care, and primary care centers, are responsible for providing basic medical treatment, prevention and rehabilitation to the whole population, irrespective of illness, age and patient group. However, the number of primary care physicians is lower in Sweden than in many other countries, both in relation to the population and in relation to the total number of physicians. Furthermore, the gate-keeping role of primary care is weaker in Sweden than in several countries with strong primary care services [26]. The 21 elected county councils run most primary health care centers, typically employing not only general practitioners (GPs) and nurses but also professionals such as physiotherapists, occupational therapists and social workers [17]. The organization of primary care into multidisciplinary group practices follows a long tradition in Sweden, but is recently becoming more common in other healthcare systems [26, 27].

The Swedish patient choice reforms and payment reforms can be seen as an attempt to strengthen the role of primary care in the healthcare system, but in particular the patient choice reforms had an ideological motivation as well [28, 29].The reforms, initiated by individual county councils and mandated by national law in 2010, are based on principles stipulating that private primary care centers have freedom of entry, and that payment should be equal for private and public centers and follow the patient’s choice of primary care center [16]. The payment comes mainly in the form of risk-adjusted capitation based on individual choice of care center, but also comprises features of fee-for service and pay-for-performance.

As county councils are entitled to design their own payment system, principles for risk-adjustment of capitation payment to primary care providers vary. A majority of the county councils have chosen to use the Care Need Index (CNI) to adjust payment, although this principle has been introduced at different times, see Table 1. The CNI is a weighted index using seven factors; the number of: children under five; inhabitants born in Europe outside the European Union, Africa, Asia, or South America; over 65 years and living alone; single parents with children under 17 years; inhabitants 1 year or older that have recently moved to the area; unemployed 16–64 years; and inhabitants 25–64 years with no more education than nine years of compulsory school [30, 31].

**Table 1:** Year of patient choice reform and introduction of risk adjustment per county council

County council	Patient choice reform	Risk-adjustment	CNI	ACG
Blekinge	2010	–	No	No
Dalarna	2010	2010	Yes	Yes
Gotland	2009	–	No	No
Gävleborg	2010	2010	Yes	No
		2013	Yes	Yes
Halland	2007	–	No	No
Jämtland	2010	2010	Yes	No
Jönköping	2010	2010	Yes	No
		2012	Yes	Yes
Kalmar	2010	2010	Yes	No
		2012	Yes	Yes
Kronoberg	2009	2011	Yes	Yes
Norrbotten	2010	2010	Yes	No
		2013	Yes	Yes
Skåne	2009	2009	Yes	Yes
Stockholm	2008	–	No	No
Södermanland	2010	2013	Yes	No
Uppsala	2009	–	No	No
Värmland	2010	2010	Yes	Yes
Västerbotten	2010	2010	Yes	No
Västernorrland	2010	2010	Yes	No
		2013	Yes	Yes
Västmanland	2008	2010	Yes	Yes
Västra Götaland	2009	2009	Yes	Yes
Örebro	2010	2010	Yes	No
Östergötland	2009	–	No	No

In parallel to risk-adjustment based on CNI, several county councils adjust capitation payment to primary care centers based on Adjusted Clinical Groups (ACG). The ACG system classifies patients by expected health care utilization in primary care based on diagnoses [32]. There is also variation between councils and over time regarding the weight of CNI in the capitated payment design (not presented in Table 1). The CNI-adjustment weight ranges between 5 and 30% across county councils. Generally speaking, county councils using CNI have either kept its adjustment weight at the same level or increased it over the years. In three county councils the CNI-adjustment kicks in over a certain threshold, specific for each council.

In the county councils not using CNI-adjustment, five use a simplified payment design with risk adjustment according to patients’ age categories. One county council has introduced a model of income-adjusted payment while another county council adjusts payments based on an assessment of special care need.

Previous studies show that the patient choice reforms in primary care have generated an overall increase in the number of primary care centers by 20% as new private centers have entered the market [33]. In addition, general access in terms of individuals using primary care services and number of visits per individual has increased. However, studies in some county councils indicate that patients with relatively high income and in relatively good health appear to gain most access [34, 35]. There is also considerable regional variation, with more private entries in populated and urban areas [33]. Thus, even if the general development indicates increased access to care, concerns have been raised about equality in access to care [17].

## Methods

### Data

For our analysis, we have built a register over all primary care centers in Sweden during 2005–2013. The register contains information about starting and closing dates, form of ownership (private/public), and exact geographical coordinates. The register has been merged with information about the payment system and other conditions for establishing new primary care centers for each county council during the same period. Information about payment systems and other conditions has been collected from county council documents, reports and surveys [36, 37], and personal communication with county council representatives.

Our unit of observation is the Small Areas for Market Statistics (SAMS). SAMS is the smallest geographic area for which Statistics Sweden produces market statistics. The classification of areas into SAMS follows the municipal sub-areas in the NYKO system (“Nyckelkodssystemet”) in larger municipalities, and electoral districts in smaller. All SAMS belong to one municipality and each municipality in turn belong to one county council during the whole period with CNI-adjusted payment. We exclude one municipality from our estimations due to its extraordinary responsibilities for health care, and lack of information about payment system used in primary care. In total we use demographic, geographic, and socioeconomic variables from Statistics Sweden for 9148 SAMS, the neighboring area of each SAMS (SAMS within the excluded municipality are included in the calculations of the neighborhood-level variables), 289 municipalities, and 21 county councils. Table 2 displays the mean, standard deviation, minimum and maximum value, and the number of unique observations per variable for year 2013.

**Table 2:** Descriptive statistics

Variables	Mean	Std dev	Min	Max	Obs
SAMS					
Private centers	0.0519	0.2496	0	4	9148
Centers	0.1273	0.3802	0	4	9148
Population	1.0462	1.3637	0	20.925	9148
CNI value	1.0896	0.5493	0	7.9394	9148
Neighborhood centers (3 km)	2.8796	4.7436	0	34	9148
Population (3 km)	23.6793	38.2292	0	287.859	9148
CNI value (3 km)	0.8814	0.6362	0	3.2465	9148
Municipality					
Density	0.1438	0.5095	0.0002	4.9165	289
Population > 65 (%)	22.7161	3.9366	13.0648	32.305	289
Mean income	244.2183	31.2225	192.4	456.3	289
Employed (%)	47.0416	2.6634	39.0451	54.1215	289
Right-wing alliance (%)	44.6813	11.0566	9.5	86.8	289
County council					
CNI	0.7143	0.4629	0	1	21
CNI&ACG	0.5238	0.5118	0	1	21
Low entry barriers	0.5714	0.5071	0	1	21
Scope of services	0.4762	0.5118	0	1	21
High capitation share	0.2857	0.4629	0	1	21
Cost responsibility	0.7619	0.4364	0	1	21

Note: Private centers, Centers, Neighborhood centers measure the number of full year equivalent centers, i.e. if there is one center active a whole year, the value is 1. Population and Population (3 km) are measured in thousands of inhabitants. Density is measured in thousands of inhabitants per km^2^, and Mean income in thousands of SEK per person over 16 years of age in 2013 prices

Our primary dependent variable, *Private centers*, is measured per SAMS in full year equivalents. That is, a center that exists all days during a year get the value 1, and one that exists half a year gets the value 0.5. To be counted as a center, it should participate in the patient choice system and be able to enroll patients. A few single GP practices operating outside of the patient choice system and small subsidiary units are therefore not included. Patients attending subsidiary units are enrolled at the parent center. A center is defined as private if a country council does not operate it. In a robustness check we have also used *Centers*, which measure the total number of private and public centers.

**Fig. 1:**
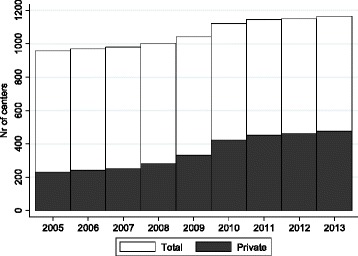
*Number of primary care centers 2005–2013*

There are far more SAMS than centers, which is reflected in the low mean values for both *Private centers* and *Centers* (0.05 and 0.13 respectively). There are also more public centers than private, although the difference has decreased substantially from 2005. Figure 1 shows the development of *Private centers* (grey bars) and *Centers* (black) during 2005–2013. The increase in total number of centers is about 20%. The increase is fully due to entry from private centers, while the number of public centers has decreased.

Our covariates are measured on the SAMS-, neighborhood-, municipality-, or county council level. On the SAMS-level, we include the number of inhabitants (*Population*¸ measured in thousands), and the SAMS-specific value on the Care Need Index (*CNI value*). We have access to all variables making up the CNI, but choose to use only *Population* and the index value itself, as all other variables are highly correlated with each other, or with the CNI.

Certain SAMS have very few or no inhabitants. In the latter case, no *CNI*-value can be calculated. We set the value of these SAMS to zero and they are therefore not included in the calculations of neighborhood variables (see below). Because of unpopulated or scarcely populated areas, we do not use the per capita number of private primary centers as the dependent variable in our baseline regressions. SAMS with very few inhabitants and one center would get a very high weight in that case, which may bias our estimations. In robustness checks, we examine how sensitive our results are to this choice.

A SAMS is in many cases too small to make up a relevant primary care center market. The decision to establish a new private center is therefore unlikely to be based solely on the characteristics of a single SAMS, but also its neighbors. However, it is difficult to define precisely the size of the relevant market, and the size may differ between parts of the country (e.g. rural and urban areas). To capture influence from the neighboring areas, we include three variables – *Centers (3 km)*, *Population (3 km)*, and *CNI (3 km)* – which are calculated as the average over SAMS whose mid-point coordinates are within three kilometers from the midpoint of the SAMS of interest. We test the sensitivity to changing this distance to 1, 5 and 10 km.

We include the following municipal-level demographic and socioeconomic variables: population density per square kilometer (*Density*), share of population over 65 years (*Population > 65*), average income in thousands of SEK and 2013 prices (*Mean income*), and the share of employed in the total population (*Employed*). We also include a variable capturing the political landscape: the joint share of votes on parties that are in general most favorably inclined to private provision of public services (the so-called *Right-wing alliance*). Privately provided elderly care is more common in municipalities with a large share of votes for these parties. It is for example possible that it has benefits to co-locate primary care centers and nursing homes for larger firms that perform both services, and we included the variable to control for this potential confounder.

On the county council level, we include seven indicator variables that characterize the payment system and the rules governing the establishment and activities of primary care centers. *CNI* takes the value 1 from the year a county council introduces CNI-adjustment. *CNI&ACG* takes the value 1 from the year a county council uses both CNI and ACG-adjusted capitation. Four county councils have only CNI-adjustment in 2013, and eleven use both CNI and ACG (no county council uses only ACG, see Table 1).

The next variable describes the enrolment principle used when a patient choice reform was introduced. *Low entry barriers* takes the value 1 that year, if a county council either let new centers automatically get a share of the patient stock, or allowed only active enrolment. The contrast group consists of county councils that only gave already existing centers a share of the patient stock, and new centers started with zero patients and only active enrolment [30]. Twelve county councils had enrolment principles that were relatively generous to new entrants by this definition.

The variable *Scope of services* indicates county councils that require primary care centers to provide more than three of the following additional services: children’s health care, maternal care, rehabilitation, home care, and podiatric care. A larger scope of services ought to imply higher start-up investment, and may therefore decrease the probability of entry. Ten county councils require a large scope of services according to this definition. Note that we do not have information about this variable for other years than 2011. We assume that the scope required have been the same for all years since the implementation of a patient choice reform.

*High capitation share* indicates county councils where capitation constitutes more than 90% of the payment to centers, a payment model used in six county councils [30]. For this variable as well, we have only information for 2011, and we make the same assumption about continuity of use. The last variable of Table 2, *Cost responsibility*, takes the value 1 in county councils where primary care centers share the responsibility for medical prescriptions to enrolled patients. Centers share this responsibility in 16 county councils in 2013. For this variable, we have information for the years 2010, 2011, and 2013. No county council changed its regulations during this period.

In the estimations, we also include a variable indicating the years when a patient choice system is in effect in a county council, *Choice reform*. As all county councils have implemented a choice system in 2013, we have omitted this variable from Table 2. We do not include any political variables on the county council level, as they are highly correlated with several of the variables describing payment and regulations. For example, the county council version of *Right-wing alliance* is highly predictive of having low entry barriers (based on own calculations).

### Empirical strategy

Our aim is to examine how CNI-adjusted capitation affects the establishment of private primary care centers in small geographical areas with low and high SES. We use a difference-in-differences (DID) type of strategy to estimate the effects. The baseline strategy compares how the number of full year equivalent private centers changes before and after county councils introduce CNI-adjustment. The comparison uses differences between county councils that do and do not introduce CNI-adjustment, and differences between SAMS with high and low CNI values within county councils. That is, the effects of CNI-adjustment on private center establishment is estimated as a “triple-difference”, using differences over time, between county councils, and between types of SAMS. For estimation purposes, we use variants of the following estimating equation:

1 $$ {y}_{st}=\alpha +{\beta}_1{CNI}_{st}+{\beta}_2{CNI}_{st}\times {HighCNI}_s+{\gamma X}_{st}+{\mu}_s+{\lambda}_t+{\varepsilon}_{st}, $$

where *y*_*st*_ is the number of full year equivalents of private primary centers in SAMS *s* in year *t*. *CNI* is an indicator taking the value 1 from the year the county council where SAMS *s* is located introduces CNI-adjusted capitation, and all years after. *CNI x HighCNI* is our main variable of interest: an interaction between *CNI* and an indicator taking the value 1 if SAMS-area *s* has a CNI-value higher than 1.0 in 2013. This value is slightly above the median (0.986) and slightly below the mean (1.06); that is, areas with CNI > 1 have relatively low socioeconomic status and relatively high expected care need. *HighCNI* is therefore time-invariant and drops out of the equation because of the inclusion of the area-specific fixed effects (its effect is captured by *μ*_*s*_). We have chosen this specification in order to keep the treatment and control groups intact over time, which facilitates tests of the parallel trends-assumption (see further discussion below). Our results are robust if we instead let *HighCNI* vary over time (results available on request).

In some regressions, we separate between county councils that use only CNI-adjusted capitation, and councils that use both CNI and ACG to adjust capitation, and then we have two indicators (*CNI* and *CNI&ACG*) and two interactions with *HighCNI*. *X*_*st*_ is a vector of time-varying covariates on SAMS-, neighborhood-, municipality-, and county council level, described in the Data section. The SAMS- and year effects are *μ*_*s*_ and *λ*_*t*_, respectively, *β*_1_, *β*_2_, and the vector *γ* are parameters to be estimated, *α* is the intercept, and *ε* is a residual term. To check how sensitive our results are to the functional form assumptions implicit in eq. (1), we use coarsened exact matching (CEM) [38].

A basic assumption that needs to hold for *β*_2_ to be interpreted as the causal effect is that the trends of the outcome variable would have been parallel in SAMS with high and low CNI values in county councils that do and do not introduce CNI-adjusted capitation, had CNI-adjustment not been introduced [39]. This assumption concerns a counterfactual scenario, and is thus not directly testable. However, if trends start to diverge already before CNI-adjusted capitation is introduced, the assumption is tenuous, at best. We perform “placebo” tests to gauge the plausibility of this assumption. In these tests, the *CNI* indicator and the interaction *CNI x HighCNI* take the value 1 one or more years before a county council actually introduces CNI-adjustment. If these estimates are small, the trends before introduction are similar, which in turn make it more likely that they would have continued to be similar, had CNI-adjusted capitation not been introduced. We also show estimations of the effects for each interval of CNI from 1.0 and up, to see how the effects vary over the range of areas with relatively high CNI.

In Additional file 1, we provide further robustness checks: we test whether our results are sensitive to using the total number of centers as dependent variable (*Centers*); a definition of the group of SAMS with high CNI values based on the values in 2005 instead of 2013; changing the definition of a SAMS neighborhood; excluding SAMS with a population under 10, 100, or 500 inhabitants, or over 10,000 inhabitants; and finally, to using a dependent variable measuring the number of private primary care centers per capita.

We report results for other variables measuring aspects of the patient choice reforms (described in the Methods section). A similar parallel trends-assumption pertains to these variables, but the estimates should be interpreted with more caution, for three main reasons: First, the variables vary only on the county council level (and over time), which makes them more likely to be correlated with other unmeasured trends that affect entry of private primary care centers. Second, we partly lack information about *Scope of services*, *High capitation share*, and *Cost responsibility* and have assumed that these variables stay the same throughout the period from the year a county council introduces its patient choice reform to 2013. Third, a few of these variables are highly correlated, in particular *Low entry barriers* and *Scope of services*, and it is therefore hard to separate out their effects.

We employ Stata 13.1 for all the statistical analyses [40]. We cluster the standard errors on the county council-level in our baseline regressions. However, there are only 21 county councils, implying that standard errors may be underestimated due to the small number of clusters-problem described in [41]. In two robustness checks, we have used a long-difference specification, and the wild-cluster bootstrap procedure suggested by [42].

## Results

Table 3 displays results from our baseline regressions. In column (1), we include only *CNI* and *CNI x HighCNI*, and no other covariates. Column (2) includes these two variables plus all covariates from Table 2. Column (3) separates between CNI-adjustment and CNI plus ACG-adjustment and excludes other covariates. Lastly, column (4) keeps the separation between CNI-adjustment and CNI- plus ACG-adjustment and adds covariates.

**Table 3:** Baseline regressions

Variables	(1)	(2)	(3)	(4)
CNI	−0.0140 *	−0.0071	−0.0167**	−0.0077*
	(0.0068)	(0.0043)	(0.0065)	(0.0039)
CNI x HighCNI	0.0223***	0.0200***	0.0185***	0.0183***
	(0.0037)	(0.0040)	(0.0043)	(0.0047)
CNI&ACG			0.0038	0.0018
			(0.0027)	(0.0025)
CNI&ACG x HighCNI			0.0052	0.0024
			(0.0046)	(0.0048)
Low entry barriers		−0.0009		− 0.0010
		(0.0063)		(0.0061)
Scope of services		−0.0117**		−0.0113**
		(0.0053)		(0.0051)
High capitation share		−0.0002		− 0.0010
		(0.0069)		(0.0070)
Cost responsibility		−0.0025		−0.0032
		(0.0061)		(0.0059)
Choice reform		0.0139**		0.0144**
		(0.0061)		(0.0060)
CNI value		0.0031		0.0031
		(0.0021)		(0.0021)
Population		0.119***		0.119***
		(0.0095)		(0.0095)
Centers (3 km)		0.0009		0.0009
		(0.0007)		(0.0007)
CNI value (3 km)		0.0191***		0.0191***
		(0.0066)		(0.0066)
Population (3 km)		0.0005		0.0005
		(0.0009)		(0.0009)
Density		0.0583***		0.0574***
		(0.0097)		(0.0096)
Population > 65		0.0052***		0.0052***
		(0.0012)		(0.0012)
Mean income		0.0002		0.0002
		(0.0003)		(0.0003)
Employed		−0.0004		−0.0002
		(0.0016)		(0.0016)
Right-wing alliance		−0.0002		−0.0002
		(0.0002)		(0.0002)
Constant	0.0252***	−0.264***	0.0252***	−0.271***
	(0.0022)	(0.0766)	(0.0022)	(0.0776)
Observations	82,332	82,332	82,332	82,332
SAMS	9148	9148	9148	9148
R^2^	0.019	0.040	0.019	0.040

*Note*: Robust standard errors clustered by county council in parentheses. ***p < 0.01, ** *p* < 0.05, *p < 0.1. All specifications contain SAMS- and year fixed effects

The estimates in columns (1) and (2) indicate that CNI-adjusted capitation increases the number of private primary centers in SAMS with relatively high CNI values, i.e. low SES areas. The coefficients on *CNI x HighCNI* are positive, significant at the 1% level, and relatively large, 0.020–0.022. The magnitude can for example be compared to the average in SAMS with CNI higher than 1.0, which is 0.064 (s.d. 0.27) over the whole period, and 0.086 (s.d. 0.32) in 2013.

Columns (3) and (4) indicate that the results are driven by CNI-adjustment, and not ACG-adjustment. The interaction *CNI&ACG x HighCNI* is positive, but small and far from significant in both columns. A potential explanation can be found in the relationship between CNI and ACG. We have only access to the ACG-index for primary care centers in one county council (Region Skåne), but in this case, ACG and CNI are negatively and significantly correlated. That is, primary care centers with enrolled patients that yield a relatively high CNI-value tend to have patients that simultaneously yield a relatively low ACG-value. Since the ACG is a more important influence on capitation in most county councils, this relationship could explain the results.

The coefficient on *CNI* is negative, and in some columns significant on the ten-percent level. This estimate is not as robustly estimated, as it is based on variation only between county councils. There are furthermore only four county councils that only use CNI-adjustment. The results indicate though that positive estimates for *CNI x HighCNI* are more driven by a change in the distribution of entry and establishment within county councils, rather than increase the total number of private primary care centers. The total marginal effect (*β*_1_ + *β*_2_) is significant on the 5% level in column (2), but not in column (1). We return to this issue below.

The patient choice reform per se contributes to an increased number of private primary care centers. The coefficient of *Choice reform* is positive and significant at the five-percent level, see column (2) and column (4). With regard to specific regulations related to the establishment of new primary care centers, only the coefficient of *Scope of services* is significant (at the five-percent level), indicating that the scope requirement acts as an disincentive for entry.

We also find a positive significant (at the one-percent level) association between the number of SAMS inhabitants (*Population*) and the number of private primary care centers. The care need of the neighboring SAMS areas appears to promote private entry, as the coefficient of *CNI value (3 km)* is positive and significant at the one-percent level. The finding indicates that private primary care centers base their location decisions on conditions in geographical areas larger than the SAMS. At the municipal level, the market size, in terms of population density (*Density*), and the demand for health care, in terms of the population share over 65 years of age (*Population > 65*), increase the number of private primary care centers significantly (at the one-percent level).

### Sensitivity analyses and extensions

This section tests the sensitivity of the results in Table 3, and examines the credibility of the parallel trends-assumption. ACG-adjustment did not seem to have an effect on private primary care center establishment. We therefore use the simpler specification, shown in column (1) and (2) of Table 3, in the estimations reported below. A first sign that the results are robust can be seen from the similarity of the results between these two specifications. That covariates do not change the estimates of *CNI x HighCNI* makes it less likely that the results are driven by omitted variables [43, 44].

In Table 4, the specifications in column (1) and (2) use CEM [38] to pre-match the SAMS on the 2005 values of the following variables: *Population*, *CNI (3 km)*, *Density and Population > 65*. These variables are significant on at least the 10% level in column (2), Table 3. *Scope of services* is also significant, but we lack values for 2005 for this variable. It is therefore not included among the matching variables. In Table 4 column (2), we also include the number of primary care centers in the SAMS in 2005 among the matching variables. We coarsen the variable by using the boundary value for the 25th, 50th, and 75th percentiles for each variable, except for the number of primary care centers which has too small variation to be partitioned into more than two parts (the boundary is 1 center). The results are very similar regardless of matching variables, and also very close to our baseline results, especially for *CNI x HighCNI*. Therefore, our estimates are relatively robust to including covariates linearly as in eq. (1).

**Table 4:** Matching and placebo estimations

	(1)	(2)	(3)	(4)
Variables	CEM 1	CEM 2	Placebo	Placebo
CNI	−0.0108*	−0.0106*	−0.0179**	− 0.0075*
	(0.005)	(0.0054)	(0.0085)	(0.0043)
CNI x HighCNI	0.0195***	0.0195***	0.0232***	0.0209***
	(0.0029)	(0.0030)	(0.0036)	(0.0040)
Placebo CNI			−0.0106**	−0.0017
			(0.0049)	(0.0011)
Placebo CNI x HighCNI			0.0045*	0.0041*
			(0.0023)	(0.0020)
Covariates	No	No	No	Yes
Observations	80,496	80,325	82,332	82,332
SAMS	8944	8925	9148	9148
R^2^	0.020	0.021	0.020	0.040

Note: Robust standard errors clustered by county council in parentheses. ***p < 0.01, ** *p* < 0.05, *p < 0.1. All specifications contain SAMS- and year fixed effects

In columns (3) and (4) we have included a “placebo” *CNI*-variable, and its interaction with *HighCNI*. These variables take the value 1 one year before a county council actually introduced CNI-adjusted capitation. If the placebo-interactions are sizeable, it is an indication that the trends differed before CNI-adjustment was introduced, which makes it less likely that parallel trends-assumption hold. Another interpretation may be that there are anticipation effects [45]; that is, private centers are aware that an adjustment will be introduced and acts upon that information. The CNI-placebo is relatively large and significant in column (3), but small and insignificant in column (4) where all covariates are included. The interaction placebo is small, but significant on the 10% level in both columns. Note also that the estimate for *CNI x HighCNI* changes very little when the placebo variables are added.

While the placebo estimations are not a source of huge concern, they do indicate a slight pre-trend difference and therefore motivate a closer look at these trends. We therefore estimate a variant of eq. (1), where we let each year, both before and after the introduction of CNI-adjusted capitation, have its own effect. This specification also allows us to examine the development of treatment effects over time after the introduction of CNI-adjusted capitation, which is of independent interest.

**Fig. 2:**
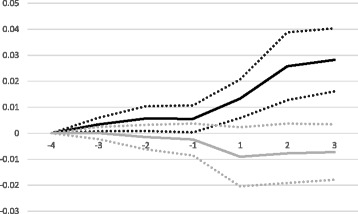
*Yearly treatment effects*

Figure 2 displays the yearly coefficients of *CNI* (grey solid line) and *CNI x HighCNI* (black solid line), and their 95% confidence interval (dotted grey and black lines respectively), spanning from three years before implementation to three years after (all lines start at zero in year four before implementation by definition). All county councils using CNI-adjusted capitation except one have at least three years before implementation and three years with CNI-adjustment in the sample period (see Table 1). The yearly effects for the county councils that have adjusted capitation according to CNI for more than three years in the sample period are included in the estimation. However, these coefficients are hard to interpret as the composition of the treatment group changes. They are therefore not included in the figure.

The figure shows a small uptick in the trend for *CNI x HighCNI* during the three placebo years, but the estimates are small in comparison to the large change in trend that is shown after the county councils introduce CNI-adjustment (year 1 in the figure). The trend is clearly increasing over these three years. The trend for *CNI* is practically flat up until the introductory year, and then turns somewhat negative but is never significant on the 5% level. These results reinforce our finding that the introduction of CNI-adjustment caused an increase in private center establishment in low SES areas, i.e. areas with high CNI.

**Fig. 3:**
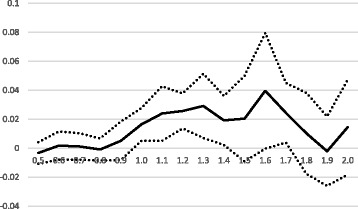
*Interval definition of high CNI*

None of the sensitivity tests reported in Additional file 1 gives us reason to reconsider our baseline results. In order to examine more closely in which areas the adjustment has an effect, we show results in Fig. 3 that indicate the effects over a larger range of CNI values. We use our baseline specification from column (2) in Table 3 (including covariates), and interact the indicator for having CNI-adjustment (*CNI*) with indicators for CNI intervals. The group of SAMS with CNI less than or equal to 0.5 serves as the reference group, and then we include interactions with indicators of intervals of 0.1 up to a CNI-value of 2.0. There are few areas with CNI > 2.0, so we include all above this boundary in one group. The figure displays the coefficients for each interaction variable (the solid black line), and their 95% confidence interval (dotted black lines).

The pattern of coefficients in the figure indicates that CNI-adjustment has a significant positive effect in SAMS with CNI values ranging from about 1.0 to about 1.8 (the interval 1.5–1.6 is not significant on the 5% level, but similar in size). Below or above these boundaries the coefficients are insignificant and mostly near zero, with a partial exception for SAMS with CNI above 2.0 where there is a slight uptick in the effect.

A question remains about the cause of the increase of private centers in high CNI areas. One potential explanation is that CNI-adjustment attracts private centers that would have otherwise established themselves in other county councils. Another explanation could be that private centers favor SAMS with relatively high CNI over those with relatively low CNI after the introduction of CNI-adjustment. Our estimation procedure uses both differences between and within county councils, so both explanations are in principle possible. As we use differences between areas and county councils, the results could in principle also be caused by changes within county councils that do not introduce CNI-adjustment. However, if we re-run our baseline model using only county councils that ever introduce CNI-adjustment our results are nearly identical. The *CNI x HighCNI* estimate is 0.021 (*p-value* < 0.001), which makes changes in non-introducing county councils a highly unlikely explanation (full results available on request).

Further evidence also points to a changed distribution within CNI-adjusting county councils as the main mechanism. First, the coefficient on the *CNI*-variable is consistently negative (although insignificant in most specifications, including our baseline). Second, running our baseline specification without the interaction *CNI x HighCNI* yields a positive, but very small and far from significant coefficient on *CNI* (0.002, *p-value* = 0.64). Third, if we run two specifications that only use differences between county councils, we find no significant differences. In the first, we contrast county councils with and without CNI-adjusted capitation but only use SAMS with CNI below 1.0. The estimate on the *CNI*-variable is negative, small and insignificant (− 0.0028, *p-value* = 0.50). In the second, we use only SAMS with a CNI equal to or higher than 1.0. In this specification the estimate is positive, but still small and insignificant (0.0046, *p*-value = 0.55). Thus, it seems like CNI-adjusted capitation mainly causes a change in the distribution of private centers *within* county councils, and make centers that would have otherwise located themselves in areas with relatively low CNI choose areas with relatively high CNI.

## Discussion

We estimate the effects of using care-need adjusted capitation on the supply of private primary care centers. Our analysis produces robust results showing that private primary care centers do react to the incentives created by risk-adjustment of capitation. Adjusted capitation significantly increases the number of private primary care centers in areas with relatively high Care Need Index (CNI). Furthermore, CNI-adjusted capitation tends to generate a larger number of private primary care centers in areas with a CNI between 1.0 and 1.8, while the effect is practically absent in areas with lower or higher values of CNI. Our analysis also shows that the care-need adjustment changes the distribution of private centers within county councils and not the total supply of private centers.

Overall, our results are good news for the governance of health care providers. The results show that economic incentives influence provider behavior in terms of location choices, and that such incentives can contribute to a more equal supply of primary care. Although the establishment of new practices tend to be more directly governed by health authorities in many other countries, our study may serve as an inspiration for countries looking for new ways to affect the spatial accessibility of primary care. The generalizability of our results also increases considering the interest in several countries in the development of group practices with an expanded role for nurses and risk-adjusted capitation payment [46, 47].

The change in the spatial distribution of private primary care centers induced by CNI-adjustment has co-existed with a large increase in the number of centers. On the one hand, our results indicate that such adjustment procedures do not affect the total number of centers. On the other hand, we do not know if we would have obtained similar results, had there not been a large increase and the primary care market had been closer to equilibrium. Furthermore, an interesting question for future research is whether the increased supply of primary care centers translates into more care and better health outcomes for the population in low SES areas. Another possibility is that the additional resources provided by the risk-adjusted payment are not allocated to services for patients, but to other purposes such as higher profits, in which case the increased supply is less likely to imply patient benefits. Individuals with low SES tend to wait longer for care or refrain from care, a behavior that may be related to e.g. financial restrictions, dissatisfaction with care, and health literacy [48–52]. Thus, for improvement in spatial accessibility to translate into increased service, additional efforts on the part of the primary care center may be required, such as outreach activities and more focus on preventive care. However, the CNI-adjustment per se does not incentivize primary care centers to exert such efforts. More evidence of how risk-adjusted payment influences service provision and in turn population health would shed light on the matter.

### Limitations

The county councils differ in the number of years they have used CNI-adjusted capitation, and we have relatively few pre-reform periods in some county councils, and few post-reform periods in others. Our results are therefore a mix of longer run and shorter run effects, and we can only estimate yearly effects for three years after introduction.

Our analysis generates relatively small R-squared values; our most developed specification explains 4% of the variation. We believe the main reason is that we lack information about some variables that determine the precise location of private primary care centers. For example, we do not have access to data about rent levels or the availability of venues suitable for primary care centers in different areas. However, this seems unlikely to be a major problem for our estimates, as the introduction of CNI-adjustment is not connected to neither levels nor changes of such variables and we detect very small differences before the introduction (as evidenced by our placebo estimates in Table 4 and Fig. 2). That is, if the development of the omitted variables is different in county councils that use CNI compared with those that do not, or in low SES areas compared with high SES areas, we would expect to see larger placebo estimates. However, we cannot rule out the possibility that there is some omitted variable or alternative specification that would explain the location choices of primary care centers better, and make the CNI-explanation less plausible.

Our analysis contains many covariates related to the various regulations of the patient choice reforms, enabling us to consider the impacts of regulatory (dis)incentives for the entry of private primary care centers in a local market. However, as noted in the Methods section, the covariates are often highly correlated with each other, making the interpretation of the estimates difficult. On the other hand, they function well as controls.

As a final point, we want to stress that our findings describe an average effect for all county councils that use CNI-adjustment. The effect is likely to vary between individual county councils, depending on the design and level of care-need adjusted capitation. In a recent report we perform a case study of three county councils, and observe that areas with very high care need attract considerably more private primary care centers in the county council that uses a high threshold value over which CNI-adjusted capitation kicks in [53]. Unfortunately, the number of county councils is small, and the variation in thresholds and other design features is relatively idiosyncratic. It is therefore difficult to examine statistically if the effects are heterogeneous.

## Conclusions

Risk-adjusted capitation based on the Care Need Index increases the supply of private primary care centers in areas with unfavorable socioeconomic characteristics. More generally, this result indicates that risk-adjusted capitation can significantly affect private providers’ location decisions. Further research is needed to analyse if these results can be generalised to other contexts, and to understand better how payment designs can best alleviate inequitable access to health care.

## Additional file

Additional file 1: Additional sensitivity tests. (DOCX 31 kb)

## Abbreviations

ACG: Adjusted Clinical Group
CEM: Coarsened exact matching
CNI: Care Need Index
DID: Difference-in-differences
GP: General practitioner
SAMS: Small Areas for Market Statistics
SES: Socioeconomic status
