# Supplementary material for: Does risk-adjusted payment influence primary care providers’ decision on where to set up practices?
Source: BMC Health Serv Res. 2018 Mar 14;18:179. doi: 10.1186/s12913-018-2983-3 (PMC5853067; doi:10.1186/s12913-018-2983-3)
Supplement: Supplementary file 1 — Additional sensitivity tests. (DOCX 31 kb) [file 12913_2018_2983_MOESM1_ESM.docx]

**Appendix: Additional sensitivity tests**

This appendix shows the results for sensitivity tests briefly reported, but not shown, in the main text. We use the specification in column (2) of Table 2 as our baseline specification, and begin by discussing our main estimate, the coefficient on *CNI x HighCNI*. Column (1) of Table A.1 shows the results from a specification with total number of centers (*Centers*) as the dependent variable. The coefficient is somewhat smaller, 0.0145, than in the baseline specification but still significant on the 1 percent level. The estimate is again similar in column (2), but smaller (0.0137), where we define the group of SAMS with high CNI-index after 2005 year’s values. In columns (3)-(5), there are only minimal changes to the results when we change the definition of a SAMS neighbourhood to from 3 km to 1, 5, and 10 km, respectively.

The estimates are not sensitive to excluding SAMS with a population under 500 inhabitants (or 10 or 100, but we omit these results for brevity), or if we exclude SAMS with a population over 10,000 inhabitants. The coefficient on *CNI x HighCNI*, shown in column (8), is still highly significant if we exclude SAMS with less than 100 inhabitants, and use a dependent variable measuring the number of private primary care centers per capita. Lastly, we examine if our standard errors are underestimated due to the small number of clusters. We have estimated a “long difference” specification where all variable values are calculated as the difference between 2005 and 2013. The coefficient of *CNI x HighCNI* in this specification is larger than in the baseline specification (0.025) and significant (*p* < 0.001). Similarly, the estimate is still significant (*p* < 0.001) when we use the wild bootstrap procedure to calculate the *p*-value.

The estimates for CNI are stable in terms of sign, and reasonably stable in terms of magnitude in all ten specifications. The significance varies widely though. In terms of significance however, we are most confident of the standard errors in columns (9) and (10). There, the estimates are not significant on conventional levels of significance and in line with our baseline specification.

**Table A1. Additional sensitivity tests**

| Panel A | | | | | |
| --- | --- | --- | --- | --- | --- |
|  | (1) | (2) | (3) | (4) | (5) |
| Variables | Centers | CNI 2005 | 1 km | 5 km | 10 km |
|  |  |  |  |  |  |
| *CNI* | -0.00502 | -0.00451 | -0.00755* | -0.00715 | -0.00834** |
|  | (0.00398) | (0.00468) | (0.00413) | (0.00435) | (.00398) |
| *CNI x HighCNI* | 0.0145*** | 0.0137*** | 0.0208*** | 0.0203*** | 0.0211*** |
|  | (0.00424) | (0.00364) | (0.00393) | (0.00420) | (0.00389) |
|  |  |  |  |  |  |
| Covariates | Yes | Yes | Yes | Yes | Yes |
| Observations | 82,332 | 82,332 | 82,332 | 82,332 | 82,332 |
| SAMS | 9,148 | 9,148 | 9,148 | 9,148 | 9,148 |
| *R^2^* | 0.029 | 0.039 | 0.040 | 0.040 | 0.040 |
| Panel B | | | | | |
|  | (6) | (7) | (8) | (9) | (10) |
| Variables | Pop > 500 | Pop < 10,000 | Per capita | Long difference | Wild bootstrap |
|  |  |  |  |  |  |
| *CNI* | -0.00818 | -0.00891** | -0.00931*** | -0.00479 | -0.00712 |
|  | (0.00628) | (0.00403) | (0.00281) | (0.00490) | (*p* = 0.168) |
| *CNI x HighCNI* | 0.0225*** | 0.0192*** | 0.0195*** | 0.0254*** | 0.0200*** |
|  | (0.00505) | (0.00357) | (0.00278) | (0.00481) | (*p* = 0.000) |
|  |  |  |  |  |  |
| Covariates | Yes | Yes | Yes | Yes | Yes |
| Observations | 50,289 | 82,039 | 72,312 | 9,148 | 82,332 |
| SAMS | 5,737 | 9,119 | 8,131 | 9,148 | 9,148 |
| *R^2^* | 0.048 | 0.026 | 0.012 | 0.064 | 0.040 |

*Note*: Robust standard errors clustered by county council in parentheses, except in column (10) where we use the wild bootstrap (Cameron et al., 2008) to calculate a p-value. ***p < 0.01, ** p < 0.05, *p < 0.1. All specifications contain SAMS- and year fixed effects.
